# Imaging the tumour microenvironment in rectal cancer: Decline in tumour blood flow during radiotherapy predicts good outcome

**DOI:** 10.1016/j.phro.2023.100417

**Published:** 2023-01-23

**Authors:** Kine Mari Bakke, Sebastian Meltzer, Endre Grøvik, Anne Negård, Stein Harald Holmedal, Lars Tore Gyland Mikalsen, Arne Engebret Færden, Lars Gustav Lyckander, Frida Marie Ihle Julbø, Atle Bjørnerud, Kjell-Inge Gjesdal, Anne Hansen Ree, Kathrine Røe Redalen

**Affiliations:** a Department of Oncology, Akershus University Hospital, Lørenskog, Norway; b Møre and Romsdal Hospital Trust, Ålesund; c Department of Physics, Norwegian University of Science and Technology, Trondheim, Norway; d Department of Radiology, Akershus University Hospital, Lørenskog, Norway; e Institute of Clinical Medicine, University of Oslo, Oslo, Norway; f Department of Diagnostic Physics, Division of Radiology and Nuclear Medicine, Oslo University Hospital, Oslo, Norway; g Department of Life Sciences and Health, Oslo Metropolitan University, Oslo, Norway; h Department of Digestive Surgery, Akershus University Hospital, Lørenskog, Norway; i Department of Pathology, Akershus University Hospital, Lørenskog, Norway; j Institute for Cancer Genetics and Informatics, Oslo University Hospital, Norway; k Department of Physics, University of Oslo, Oslo, Norway; l Sunnmøre MR-klinikk, Ålesund, Norway

**Keywords:** Perfusion, Blood flow, MRI, Rectal cancer, Response assessment

## Abstract

**Background and purpose:**

Measuring rectal tumour response to radiation is pivotal to restaging patients and for possibly stratification to a watch-and-wait strategy. Recognizing the importance of the tumour microenvironment, we investigated a less explored quantitative imaging marker assessing tumour blood flow (BF) for its potential to predict overall survival (OS).

**Materials and methods:**

24 rectal cancer patients given curative-intent neoadjuvant radiotherapy underwent a multi-echo dynamic magnetic resonance imaging (MRI) sequence with gadolinium contrast for quantification of tumour BF before either 25x2 Gy (n = 18) with concomitant chemotherapy or 5x5 Gy (n = 6). CD34 staining of excised tumour tissue was performed and baseline blood samples were analysed for lactate dehydrogenase (LDH) and angiopoietin-2 (ANGPT-2). Tumour volumes were measured before and after treatment. After subsequent surgery, ypTN scoring assessed tumour response. Cox regression for 5-year OS analysis and *t*-test for group comparisons were performed.

**Results:**

The change in tumour BF (ΔBF) during neoadjuvant radiotherapy was a significant marker of OS, whereas tumour stage and volume were not related to OS. All patients with >20 % decline in BF were long-term survivors. Separating cases in two groups based on ΔBF revealed that patients with increase or a low decrease had higher baseline LDH (p = 0.032) and ANGPT-2 (p = 0.028) levels.

**Conclusion:**

MRI-assessed tumour ΔBF during neoadjuvant treatment is a significant predictor of OS in rectal cancer patients, making ΔBF a potential quantitative imaging biomarker for treatment stratification. Blood LDH and ANGPT-2 indicate that non-responding tumours may have a hypoxic microenvironment resistant to radiotherapy.

## Highlights

• Tumour perfusion was measured by magnetic resonance imaging in rectal cancer.
• A decline in tumour perfusion during radiotherapy predicted favourable outcome.
• The perfusion measurements were supported by CD34 staining of tumour specimens.

## Graphical abstract

**Figure:**
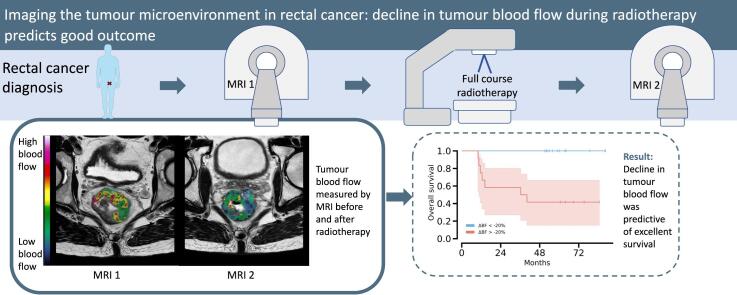

## 1 Introduction

Mapping the tumour response to radiotherapy has been a great interest of research for many years. Due to this effort, it has become evident that the tumour response to radiation is inevitably linked to the tumour microenvironment (TME) [1]. While the TME consists of several different cell types, such as immune cells, tumour-associated macrophages, and fibroblasts, it is probably the endothelial cells that have been the most studied to date [2]. The endothelial cells, making up the vascular architecture and thus enabling blood perfusion and delivery of oxygen and nutrients, are a crucial element in determining the nature of the TME. Endothelial cells within the TME are also characterized by rapid proliferation and therefore highly radiosensitive [3], [4].

Radiation is a pivotal component of the neoadjuvant therapy in conventional clinical treatment of locally advanced rectal cancer (LARC) as a way of facilitating surgical removal, either by downsizing or downstaging the tumour [5]. LARC patients may also be at considerable risk of metastasis of tumour cells to distant organs. High resolution morphological MRI is the standard tool for staging rectal cancer, including restaging tumours after radiation [6]. Restaging of rectal cancer is essential for surgical planning, as good responders may be eligible for less invasive surgery. A certain percentage of patients achieve pathological complete response [7], making them eligible for the organ-preserving watch-and-wait strategy [8], [9].

To characterize and understand the biological response of the TME to radiation, mapping the tumour with a functional imaging technique is of interest. In particular, functional MRI can give information about the perfusion of the tumour, indirectly assessing the response of the endothelial cells of the TME. We recently showed that perfusion, or blood flow (BF), from dynamic susceptibility contrast (DSC) MRI was a prognostic factor at baseline for rectal cancer patients of all stages [10], whereas parameters derived from dynamic contrast enhanced MRI were not. DSC MRI is an established method for measuring cerebral blood flow in cases of acute ischemic stroke [11], [12], but the method is not commonly used to measure perfusion in solid tumours.

In the present study we used the same DSC MRI signal to quantify BF of the TME before and after radiotherapy of rectal cancer patients. In addition, we obtained serum samples to analyse for angiogenic factors and performed immunohistochemistry with the endothelial marker CD34 on the surgical specimens. Our main hypothesis was that the BF quantified from DSC MRI before and after radiotherapy could be a determining factor in patient prognosis, and as such may be a future candidate for aiding the stratification of patients to a watch-and-wait strategy.

## 2 Methods

### 2.1 Study participants

This study was part of a prospective observational study (ClinicalTrials.gov: NCT01816607), where 192 patients with suspected rectal cancer were enrolled between October 2013 and December 2017 at Akershus University Hospital. Eligible patients had histologically confirmed rectal adenocarcinoma, were older than 18 years and had no prior rectal cancer treatment. In this sub-study, patients that received neoadjuvant treatment before surgery were analysed, the clinical ratification for neoadjuvant treatment was determined by a multidisciplinary team by applying the 2013 ESMO Clinical Practice Guidelines [13] in accordance with the imaging updates in the 2017 version [14]. Of the 192 enrolled patients 67 were eligible for neoadjuvant treatment. Of these, 30 patients underwent the study MRI both before and after treatment, while 37 had follow-up MRI at another cancer centre. The follow-up MRI was performed after completion of the neoadjuvant treatment. Of the remaining 30 patients, 5 had too poor quality of the MRI either before or after treatment, while 1 patient had no residual tumour visible on the post-MRI left for analysis. All 24 patients were given curative-intent treatment. Table 1 summarizes the patient characteristics. Approval for this study was obtained from the Institutional Review Board and the Regional Committee for Medical and Health Research Ethics. The study was performed in accordance with the Helsinki Declaration, written informed consent was obtained from all participants.

**Table 1:** Patient characteristics.

Parameter	Value*
No. of participants (women/men)	24(7/17)
Age (years)	63 ± 12

*TNM:*	
T2/T3/T4	1/17/6 (4 %/70 %/25 %)
N0/N1/N2	7/11/6 (29 %/46 %/25 %)
M0/M1	21/3 (88 %/13 %)

*Stage (AJCC):*	
1	2 (8 %)
2	4 (17 %)
3	15 (63 %)
4 (resectable liver metastasis)	3 (13 %)

*Treatment:*	
Short-course radiotherapy (5 × 5 Gy)	6 (25 %)
Long-course radiotherapy (2 × 25 Gy) with concomitant chemotherapy	18 (75 %)

*Timing:*	
Weeks from first MR to radiotherapy	4.5 (1)
Weeks from end of radiotherapy to follow-up MRI	5 (1)
Weeks from end of radiotherapy to surgery	8.5 (1.5)

*ypT:*	
0/1/2/3/4	5/2/3/12/2 (21 %/8%/13 %/50 %/8%)
ypN: 0/1/2	15/5/4 (63 %/21 %/17 %)

*Tumour volume:*	
Volume_pre_ (cm^3^)	30 ± 25
Volume_post_ (cm^3^)	15 ± 9
ΔVolume (%)	− 52 ± 22

*Blood flow (BF):*	
BF_pre_ (ml/min/100 g)	104 ± 26
BF_post_ (ml/min/100 g)	89 ± 37
ΔBF (%)	−11 ± 37

*Diseased:*	
Women	1 (14 %)**
Men	6 (35 %)

*Quantity (percentage) or mean (SD) as appropriate.

**Percentage of women/men in study.

### 2.2 MRI

All patients received diagnostic work-up MRI consisting of fast-spin echo T_2_-weighted images and a diffusion weighted sequence. The T_2_-weighted images were obtained in the sagittal and transversal direction, as well as perpendicular to the tumour axis (axial, or oblique-axial), with repetition time (TR) = 2820–3040 ms, echo time (TE) 80 ms, slice thickness = 2.5 mm, number of averages = 6 and echo train length = 20. In addition, the patients underwent a multi-echo dynamic contrast enhanced sequence with an exogenous contrast agent (Dotarem 279.3 mg/mL, Guerbet Roissy, France). This sequence was obtained as a 3D multishot *EPI* sequence with three echoes, echo times = 4.6, 13.9, 23.2 ms, repetition time = 39 ms, and flip angle 39°, time resolution varied between 1.9 and 2.5 s. The acquired matrix size was 92 × 90 over a 180 × 180 field of view and a 10 mm slice thickness with the image plane the same as the T_2_-weighted axial or oblique-axial image. The MRI was performed on a Philips Achieva 1.5 T system (Philips Healthcare, Best, the Netherlands). More details of the MRI sequences have been published earlier [15].

### 2.3 Image postprocessing

From the multi-echo dynamic contrast sequence, we extracted the T_2_*-weighted signal, for a DSC analysis, to estimate the BF. We used the standard deconvolution approach [11], with individual arterial input function (AIF), found with a semi-automatic algorithm [16]. Much attention was directed on finding a reproducible and stable AIF for each examination, making sure that the same size and shape of the AIF could be found in several slices and areas of the images. Image processing of the dynamic data was done in NordicICE (NordicNeuroLab, Bergen, Norway). Details of the postprocessing have been published earlier [15].

Tumour regions were drawn separately by two radiologists with 14 and 7 years of experience on anatomical T_2_-weighted data, before they were semi-automatically co-registered to the functional data. Segmentation uncertainty between the radiologist were low as reported previously [10]. The tumour volumes were estimated from these delineations, accounting for the slice gap between the images. The change in volume was calculated as Δvolume = 100 * (volume_post_ − volume_pre_)/volume_pre_. The change in BF, ΔBF, was calculated similarly.

### 2.4 Immunohistochemistry

Of the patients included in this study, 11 had tumours prepared for immunohistochemistry. Tissue sections (4 μm thickness) were deparaffinized and hydrated followed by heat-induced epitope retrieval (20 min at 97° C) in Dako (Carpinteria, CA) PT-link with target retrieval solution with high pH (code K8004). Incubation time was 30 and 20 min for the first and secondary antibody, respectively. Counterstaining was performed with Hagens hematoxylin (diluted 1:4) for 5 min. Staining with CD34 (mouse monoclonal antibody, clone QBEnd/10, Nordic BioSite, Sweden) was performed with the Autostainer Link (Dako), using the Dako Envision Flex Code 8000 visualization kit. The slides were scanned using the Aperio Scanscope AT with a 0.5 μm/pixel resolution.

Tumour regions were delineated with the guidance of an experienced pathologist. The stained fractions from each tumour slide were calculated using an automated and adapted Matlab script [15].

### 2.5 Serological factors

As part of the routine blood analyses at time of diagnosis, C-reactive protein, carcinoembryonic antigen and lactate dehydrogenase (LDH) were measured. In addition, vascular endothelial growth factor A and angiopoietin 2 (ANGPT-2) were measured in the study biobank samples with a multiplex immunoassay (Luminex; R&D Systems, Minneapolis, Minn). Blood samples were taken on the same date as the first MRI.

### 2.6 Clinical data

T- and *N*-stage after radiotherapy (ypTN) was assessed by experienced pathologists as part of the clinical routine. Overall survival (OS) was calculated from study enrolment to death or patient censoring. Median follow-up time was 64 months (range 51–88) when the data was last censored, January 31st, 2022.

### 2.7 Statistical analysis

To check for differences between groups we performed Mann-Whitney U-tests. For survival analysis we used a univariate Cox regression, treating volume, BF, tumour stage and ypT as continuous and RT fractionation as categorical. Spearman’s correlation analysis was used for correlation. The statistically significant results for OS were visualized with a Kaplan-Meier plot. Results were deemed significant at p < 0.05. All statistical procedures were performed with SPSS, version 27 (IBM, Armonk, NY).

## 3 Results

**Fig. 1:**
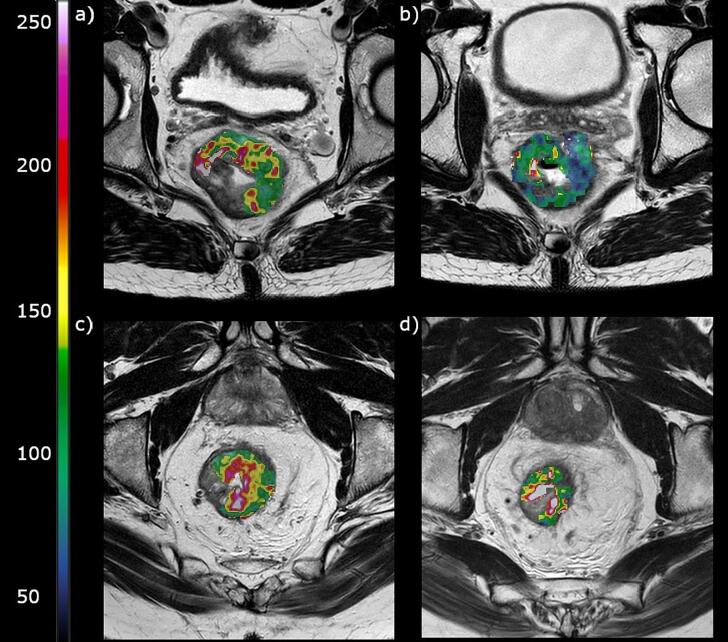
T_2_ weighted images with the corresponding tumour blood flow (BF) maps as an overlay. Patient 1 had a tumour BF at baseline (a) of 93 ml/min/100 g, and a decrease (-ΔBF) of 31 % after therapy (b). The patient had a 25 % reduction in tumour volume during treatment. The patient was alive without local recurrence or metastasis after 38 months. Patient 2 had a tumour BF at baseline (c) of 111 ml/min/100 g, and an increase of 30 % after therapy (d). The patient had a 70 % reduction in tumour volume during treatment. The patient had a local recurrence after 10 months and died 2 months thereafter.

**Fig. 2:**
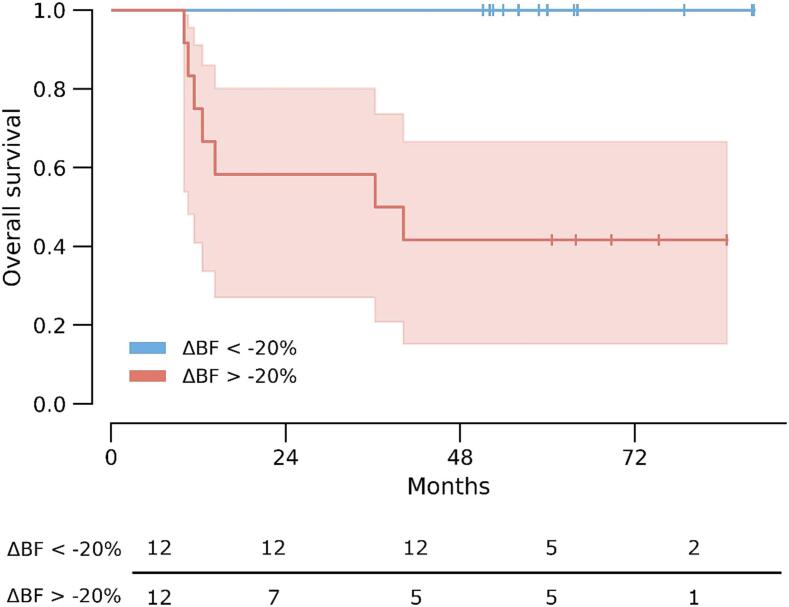
The Kaplan-Meier plot shows the difference in overall survival between the patients with a decrease in tumour blood flow (ΔBF) of 20 % or more (blue line) and the patients with less or no decrease in ΔBF (red line), p = 0.002 with a Mantel-Cox test. Patients at risk are indicated below.

Patients had a mean change in BF during radiotherapy, ΔBF, of −11 %, however, with substantial differences. Six patients had an increase in BF varying from 9 % to 77 %, while 18 patients had a decrease in BF varying from −7% to −52 %. ΔBF was significantly associated with OS, while disease stage, ypT, radiotherapy fractionation and tumour volume were not associated with OS (Table 2). Cox regression showed that less decrease in ΔBF was associated with poor OS (hazard ratio = 1.023, confidence interval = 1.005 – 1.042, p = 0.013). Fig. 1 shows examples of T_2_-weighted and BF images of two patients with different outcomes. A Kaplan-Meier plot with the median cut-off at ΔBF = −20 % is shown in Fig. 2.

**Table 2:** Uni-variate Cox regression for overall survival.

Parameter	Hazard ratio for impaired OS	p-value
Tumour stage*	1.08 [0.40–2.95]	0.88
ypT score of the surgical specimen	1.54 [0.76–3.12]	0.23
RT fractionation (25 × 2 Gy versus 5 × 5 Gy)	2.24 [0.50–9.90]	0.29
Volume_pre_	1.01 [0.99–1.04]	0.43
Volume_post_	1.01 [0.98–1.05]	0.42
ΔVolume	1.03 [0.99–1.07]	0.15
BF_pre_	0.96 [0.92–1.00]	0.05
BF_post_	1.02 [1.00–1.04]	0.11
ΔBF	1.02 [1.01–1.04]	0.01**

*AJCC.

** Bold values denote statistical significance at the p < 0.05 level.

Stratifying patients according to sex suggested that this pattern was more prominent in men, Supplementary Fig. 1a. Differences in treatment regimen had no effect on this result, Supplementary Fig. 1b. Supplementary Figs. 2 and 3 shows Kaplan-Meier plots for the different groups. In a multivariate Cox regression with ΔBF and sex only ΔBF came out as significant with hazard ratio = 1.024, confidence interval = 1.006–1.043, p = 0.010, and hazard ratio = 4.617, confidence interval = 0.527–40.463, p = 0.167 for sex.

**Fig. 3:**
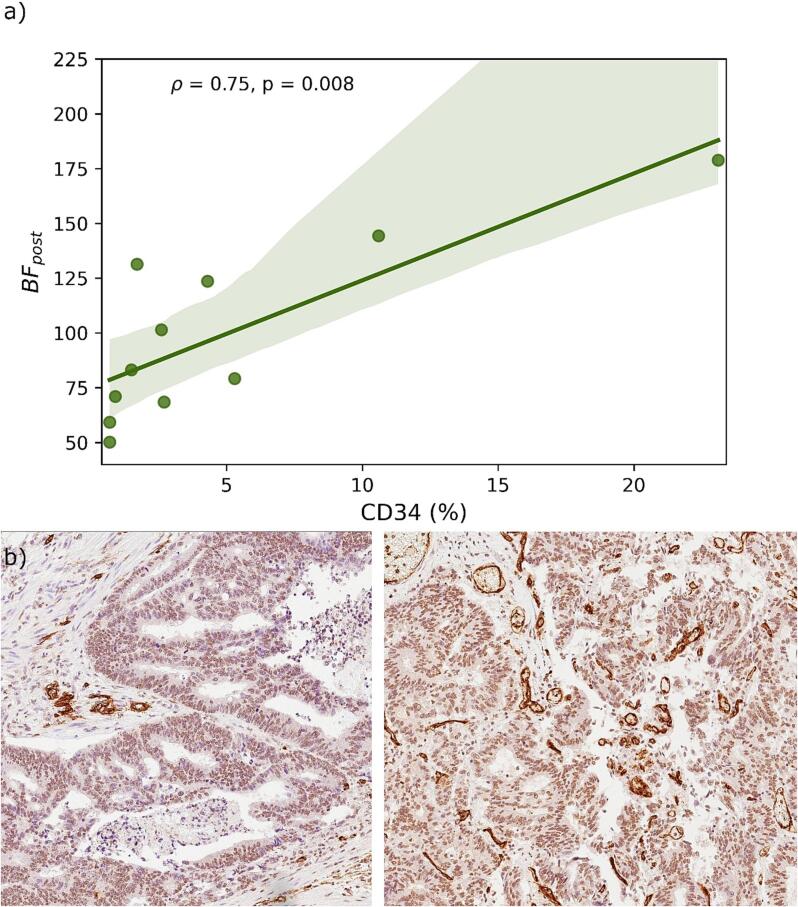
a) Correlation between the mri-assessed tumourblood flow (BF) after treatment and the percentage endothelial marker CD34 staining in the excised tumour specimen. Spearman’s orrelation coefficient (ρ) and p-value are indicated. b) CD34 staining of two different patients with 1.5% staining (left) and 10.6% staining (right). CD34 highlights the endothelial cells in blood vessels, showing strong staining. There is some weak unspecific background staining in the epithelial cells.

Percentage of CD34 staining was strongly correlated to BF after radiation (rho = 0.78, p = 0.004), Fig. 3. No other parameters were correlated to CD34 percentage.

**Fig. 4:**
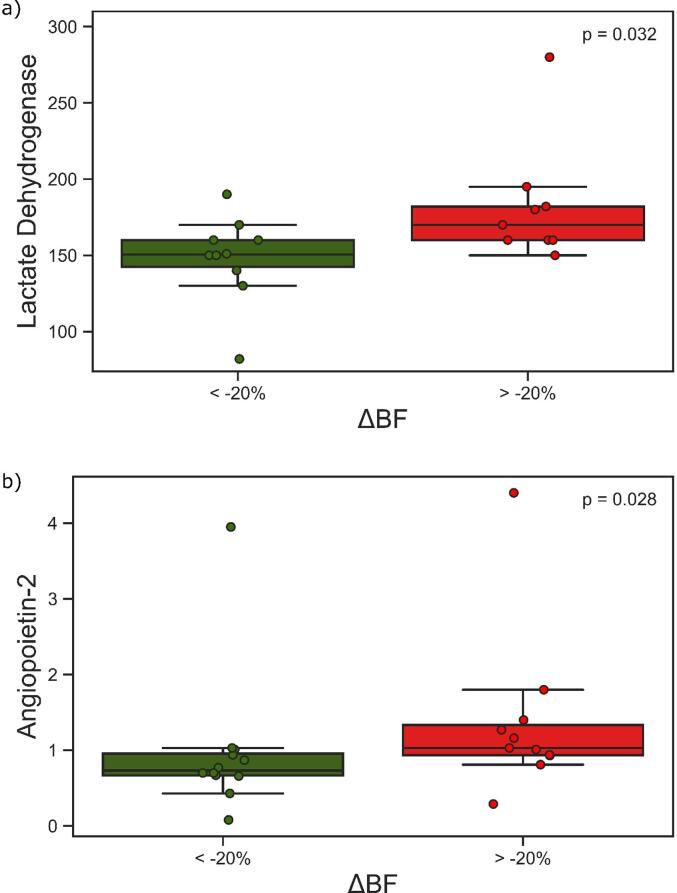
The differences in lactate dehydrogenase (a) and angiopoietin-2 (b) between the group having more than 20% decrease in tumour blood flow (ΔBF) and the group having less or no decrease.

Separating cases in two groups based on ΔBF (with ΔBF = -20 % as a cut-off) revealed that patients with increase or a low decrease had higher baseline levels of LDH (p = 0.032) and ANGPT-2 (p = 0.028), Fig. 4. No other parameters were significantly different in these groups.

## 4 Discussion

In this prospective biomarker study, we show that a decrease in blood flow during radiotherapy was predictive of long-term overall survival. The decline in perfusion during radiotherapy for the patients with good outcome can probably be attributed to the death of the endothelial cells, which have been shown to be particularly radiosensitive [3], [4]. This destruction of the vascular system may lead to a reduced vascular density and increased distance between functioning vessels [2]. This will further restrict oxygen and nutrients to the tumour cells, contributing to cell death.

When separating our cases into two groups based on ΔBF, with ΔBF of −20 % identified as an optimal cut-off, the patients with an increase or a low decrease in ΔBF had higher baseline levels of LDH and ANGPT-2. The expression of both these factors has been shown to be increased under hypoxia [17], [18], indicating that these tumours were already on a hypoxic pathway, making them more resistant to radiation [19].

Restaging of tumours is routinely done on anatomical MRI, with diffusion-weighted MRI as an extra guidance. However, before the tumour has visibly disappeared from the patient, a radiation-induced change in the tumour TME must occur, that facilitates the death of the tumour cells. Viewing the anatomical extent of the tumour may fail to take into consideration this changing functionality of the irradiated TME. Indeed, we showed that the change in tumour volume was not a significant predictor of OS. We believe that the change in BF better reflects the treatment response by showing the vitality of the tissue. This might indicate that BF measurements from DSC MRI can become a useful tool to determine treatment response to radiotherapy, and possibly stratify patients to a less invasive surgery such as the watch-and-wait approach. According to the recent review by López-Campos et al. [20], the criteria for selecting rectal cancer patients for a watch-and-wait strategy are still one of the controversies that needs to be resolved, and we propose quantitative BF measurements from DSC MRI as a relevant tool for this purpose.

In accordance with the SAGER guidelines [21], we have stratified our blood flow measurements according to sex, we observe then that our significant results were only valid in men. In our earlier publication on blood flow in rectal cancer we also observed that the relationship between poor blood flow and poor prognosis were only present in the male population. While sex differences in colorectal cancer have been a much-discussed topic recently [22], there are unfortunately too few female patients in this study to expound on this issue.

The patients in this study had two different treatment regimens as six patients underwent short-course 5 × 5 Gy radiotherapy and the other 18 patients received long-course 25 × 2 Gy with concomitant chemotherapy, but this difference in treatment did not affect the results of this study.

We identified a strong correlation between percentage staining of the endothelial marker CD34 and BF after radiotherapy. This strengthens the assumption that BF measurements using DSC MRI post-radiation is feasible in rectal cancer.

While perfusion markers from DSC have been little investigated in rectal cancer, the apparent diffusion coefficient (ADC) from diffusion weighted MRI has received a lot of attention as a biomarker for radiotherapy response. ADC has shown promise as a marker for complete response, interpreted as treatment-induced cell death, but has not shown the same promise as a prognostic marker of long-term survival. In our previous work where we analysed a larger cohort, the ADC was less associated to both short- and long-term endpoints than the perfusion marker BF. We believe the BF reflects different aspects of the TME, possibly relating to the metastatic ability of the tumour, since majority of events linked to long-term survival involves metastatic development.

The major limitation of this study is the low number of patients included and the heterogeneity of the treatment the patients received. However, we show that the different treatment regimens produce the same pattern of results and is therefore most likely not a confounding factor in this case.

The low number of patients included reflects the difficult logistics of getting patients to return to the exact same scanner for the second MRI. Making MRI sequences and analysis methods that are robust enough to be applied on scanners from different vendors and at different hospitals continues to be a necessary step to enrol enough patients for robust statistical analysis, and to be able to integrate these methods into the clinical workflow.

In summary, we report results using an old quantitative DSC MRI method of measuring tumour BF, in the new setting of rectal cancer response evaluation. We show that calculating the change in BF during radiotherapy in rectal cancer patients is feasible and predicts OS. The method represents a potential tool to stratify patients to individualized follow-up after radiotherapy, such as less invasive surgery using watch-and-wait strategies.

## Declaration of Competing Interest

The authors declare that they have no known competing financial interests or personal relationships that could have appeared to influence the work reported in this paper.
