# Supplementary material for: Imaging the tumour microenvironment in rectal cancer: Decline in tumour blood flow during radiotherapy predicts good outcome
Source: Phys Imaging Radiat Oncol. 2023 Jan 23;25:100417. doi: 10.1016/j.phro.2023.100417 (PMC9883255; doi:10.1016/j.phro.2023.100417)
Supplement: Supplementary data 1 [file mmc1.docx]

Supplementary

Supplementary Figure 1:

a) Change in tumour blood flow (ΔBF) where the patients have been split by the different treatment regimens of 2x25 Gy (n = 18) with concomitant chemotherapy and 5x5 Gy (n = 6). The cut-off at -20% is indicated. b) Change in tumour BF where patients have been split by sex. The cut-off at -20% is indicated.


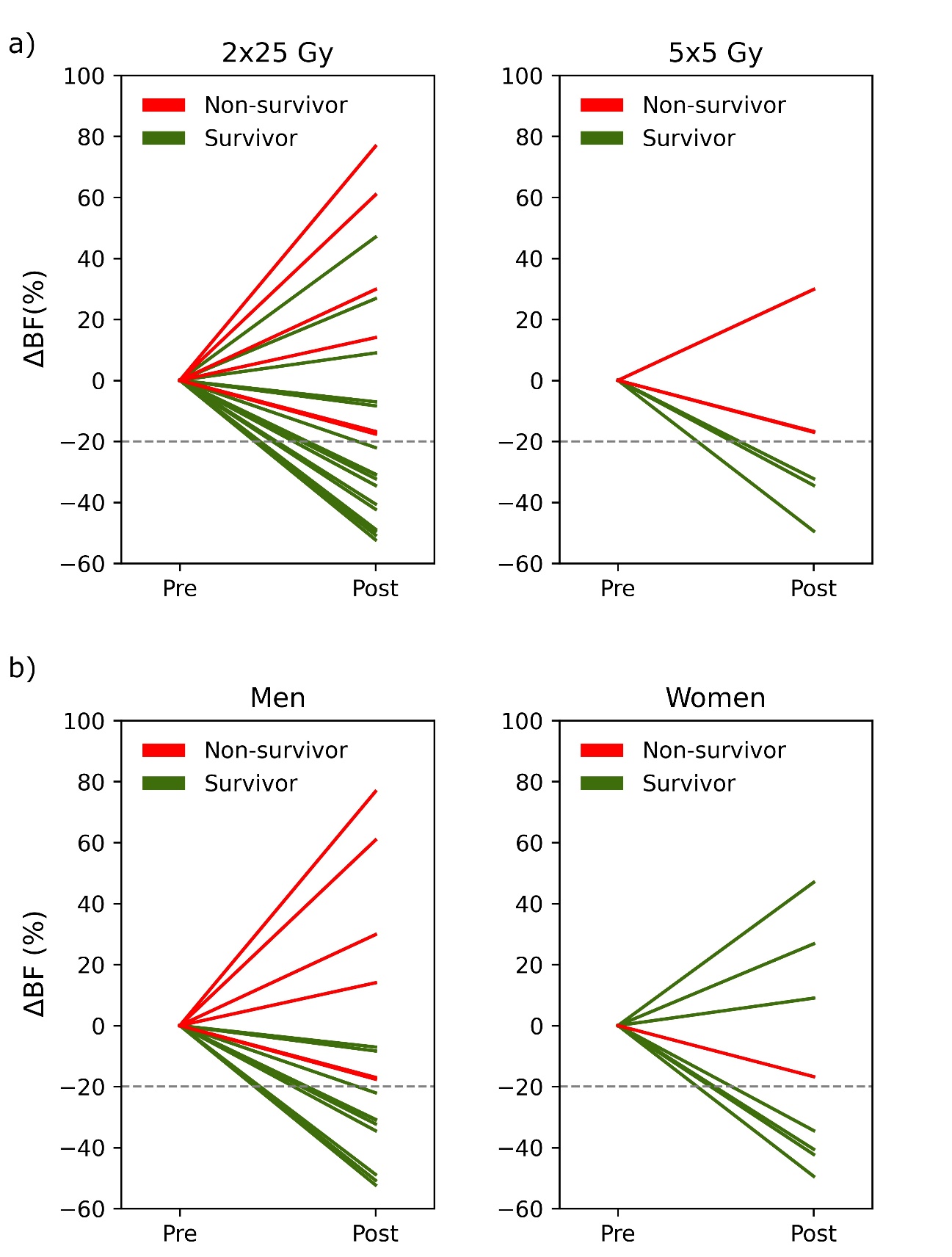


Supplementary Figure 2:

a) The Kaplan-Meier plot shows the difference in overall survival between the patients with a decrease in tumour blood flow (ΔBF) of 20% or more (blue line) and the patients with less or no decrease in ΔBF (red line) for patients receiving 25 x 5 Gy, p = 0.027 with a Mantel-Cox test. Patients at risk are indicated below. b) Patients receiving 5 x 5 Gy, p = 0.025.
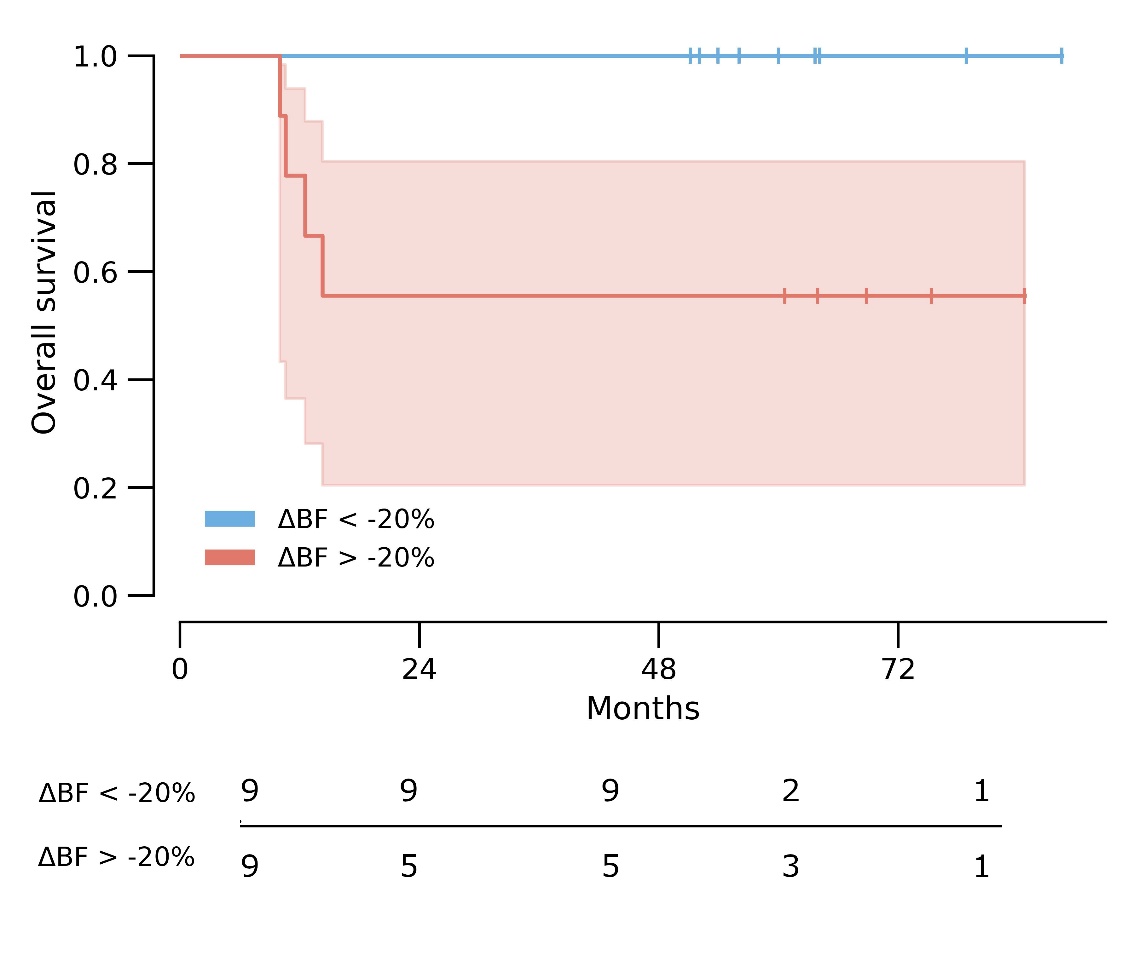

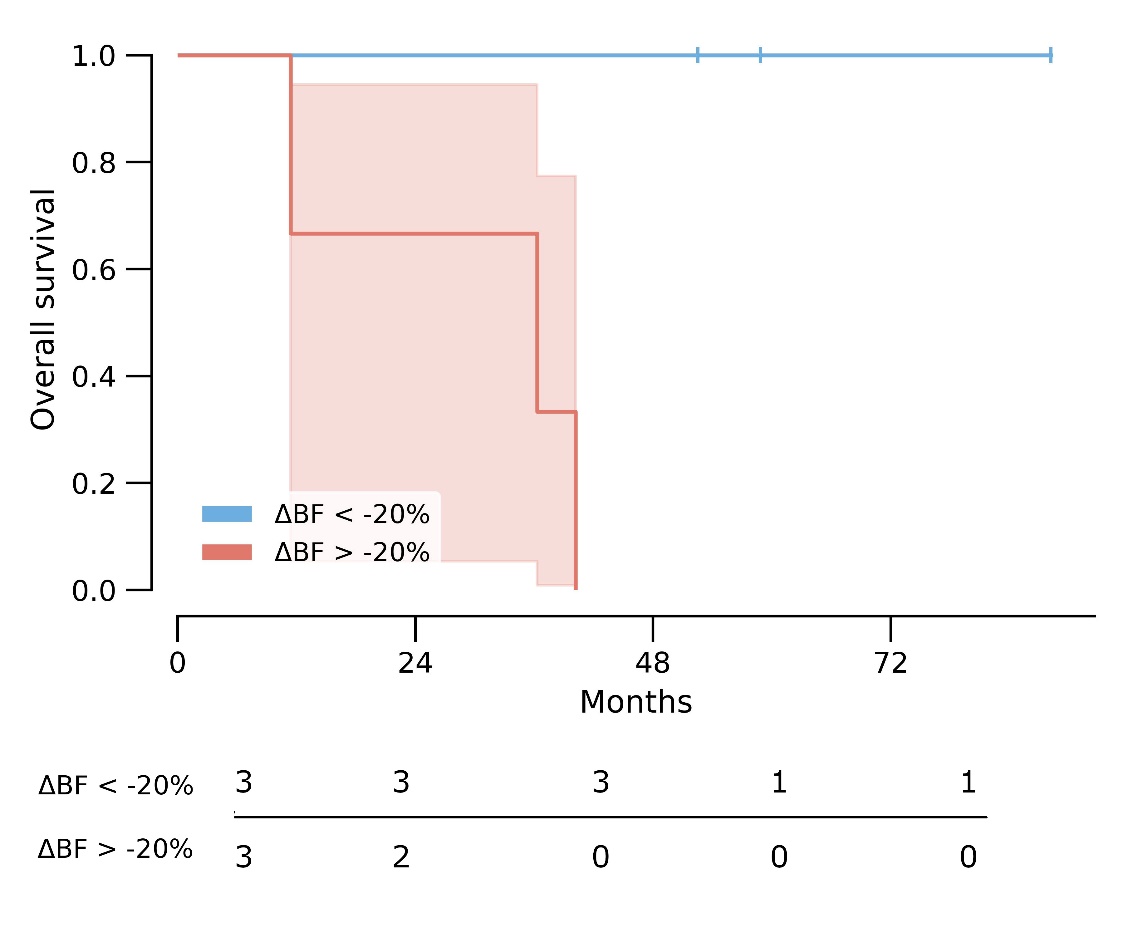


a)

b)

Supplementary Figure 3:

a) The Kaplan-Meier plot shows the difference in overall survival between the patients with a decrease in tumour blood flow (ΔBF) of 20% or more (blue line) and the patients with less or no decrease in ΔBF (red line) for females, p = 0.317 with a Mantel-Cox test. Patients at risk are indicated below. b) Males, p = 0.002.
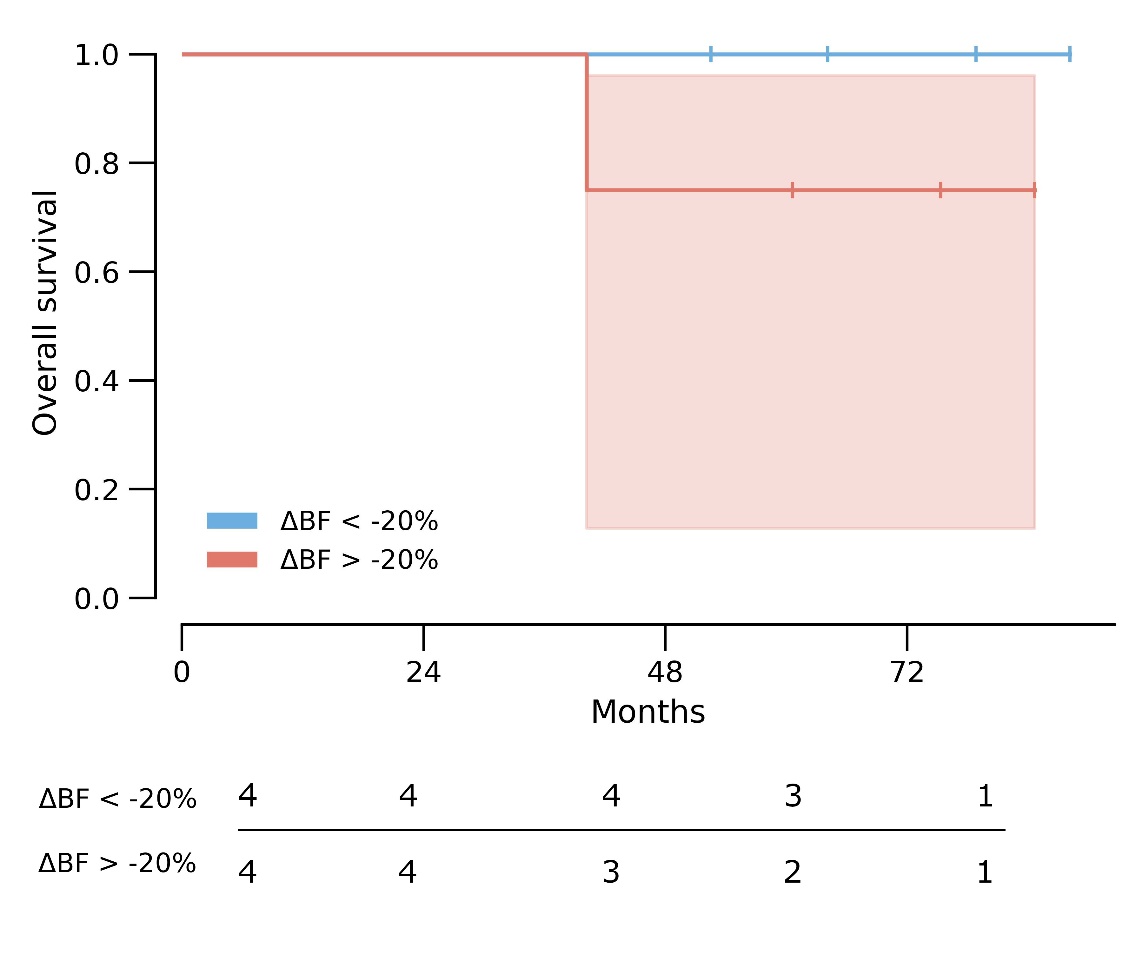

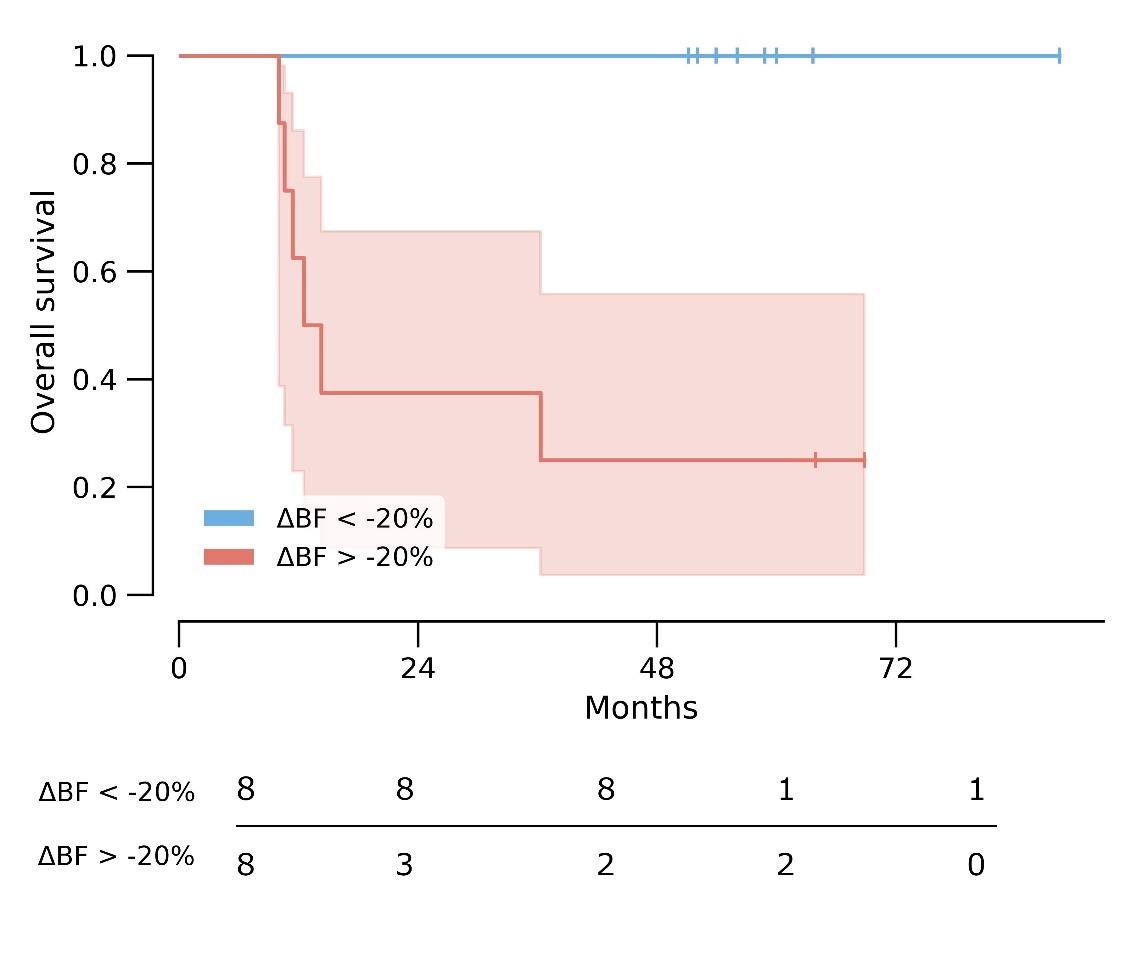


a)

b)
